# Novel Use of Joint Replacement in a Thumb Interphalangeal Joint

**DOI:** 10.1155/2019/2603098

**Published:** 2019-04-15

**Authors:** Desirae McKee, E. L. Domingo-Johnson

**Affiliations:** Department of Orthopaedic Surgery, Texas Tech University, 3601 4th Street, Mail Stop 9436, Lubbock, TX 79430-9436, USA

## Abstract

Arthrodesis of the interphalangeal (IP) joint of the thumb is widely acknowledged as the indicated treatment for trauma and osteoarthritis of this joint. Joint replacement was offered to a young patient as an alternative treatment after traumatic joint loss of the IP joint in her thumb. This allowed her to maintain motion of the joint as she did not wish to move forward with fusion. In the present case, a Humanitarian Use Device (HUD) was utilized in an off-label manner and placed into the interphalangeal joint of the patient's thumb. The patient did well postoperatively with reasonable pain-free range of motion of the interphalangeal joint with an arc of 40 degrees. We propose that joint replacement is a viable alternative to arthrodesis in select patients who do not wish to be fused. We believe this represents the first thumb IP joint replacement using a semiconstrained joint presented in the medical literature.

## 1. Introduction

The interphalangeal joint of the thumb provides the flexibility and range of motion necessary for activities that characterize modern living such as allowing for the use of a handheld cellular device as well as simple activities of daily living such as lacing up shoes. However, when the structural integrity of this joint is compromised, surgical immobilization of the joint is often required to prevent pain at the joint. Arthrodesis of the interphalangeal (IP) joint of the thumb is widely acknowledged as the indicated treatment for trauma and primary osteoarthritis of this joint [1]. Although standard treatment for interphalangeal joint trauma is IP arthrodesis, in the case of a 15-year-old female patient presenting with traumatic loss of the interphalangeal joint from a table saw, other treatment options were explored due to patient desire to avoid fusion. Small joint arthroplasty is a procedure typically reserved for use in the interphalangeal joints of the 2^nd^ through 5^th^ digits. In this procedure, the diseased or damage joint is removed and replaced with a metal/plastic or pyrocarbon implant that recreates the joint space. One other available option for small joint arthroplasty is a silicone joint implant. Given the patient's age, silicone implantation was ruled out due to the likelihood of implant breakage over time [2, 3]. Pyrocarbon implants have been used in proximal interphalangeal (PIP) arthroplasty with higher rates of survivorship shown at a long-term follow-up [4, 5]. The principle of using nonflexible material to increase longevity of a PIP joint implant was applied to the IP joint in the case we describe. In the present case, the patient's joint was traumatically damaged secondary to injury and subsequently replaced during treatment with a Humanitarian Use Device (HUD) consisting of high molecular weight polyethylene, cobalt chrome, and titanium. An HUD is a medical device which aids in the treatment or diagnosis of a disease or condition affecting less than 8,000 individuals in the United States per year [6]. Although placement of the prosthesis into the interphalangeal joint of the thumb is considered an off-label use of the HUD, we propose that our approach was the best method of treatment as it enabled this patient to retain some mobility of the joint rather than an arthrodesis which the patient did not desire. Current review of the published scientific literature did not reveal a similar case of joint replacement in the thumb although a newspaper article from the UK did comment on a silicone IP joint replacement in a thumb [7]. In that case, the patient specifically was able to regain function of the thumb for his professional requirements of being a magician.

## 2. Case Presentation

**Figure 1:**
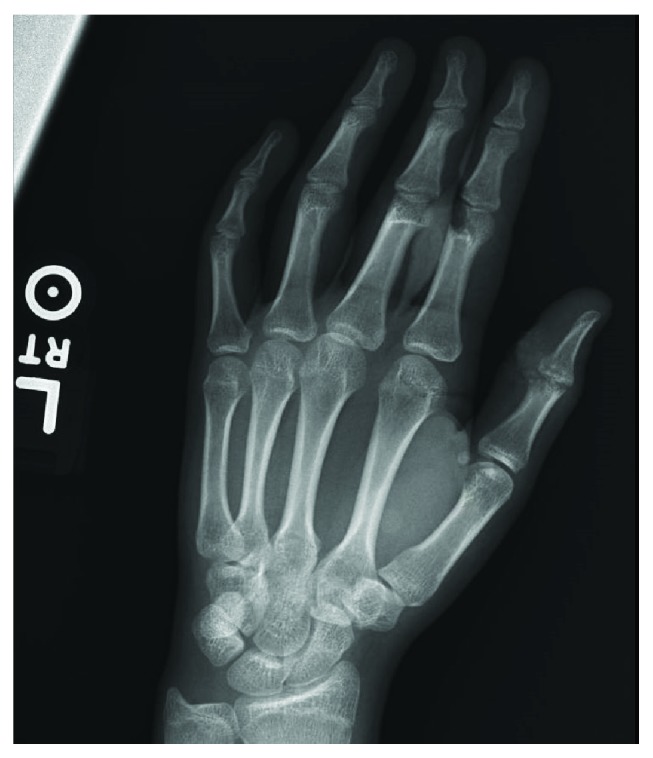
Preoperative AP view of the thumb.

**Figure 2:**
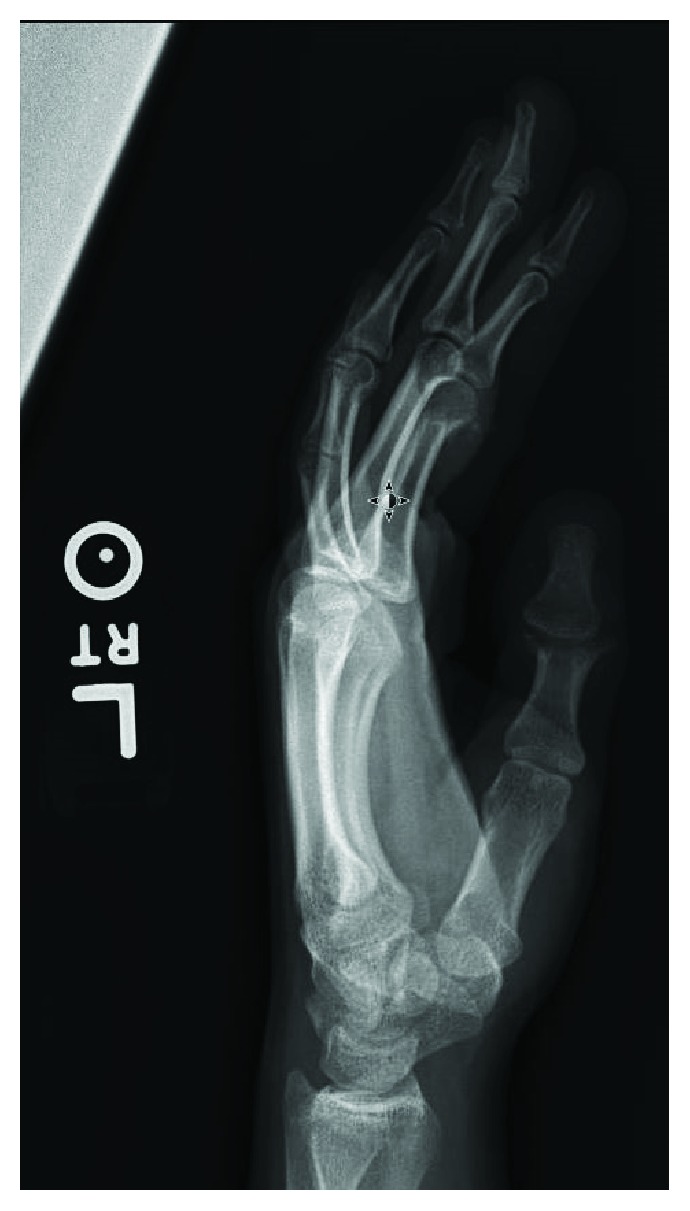
Preoperative lateral view of the thumb.

A 15-year-old Caucasian female presented with an intra-articular fracture and adjacent laceration at the distal portion of the proximal phalanx of the thumb resulting in near complete loss of the interphalangeal joint (Figures 1 and 2). The injury was incurred from a table saw accident. The patient had an initial pain level of 4/10 and was unable to flex her thumb. The patient and her family were offered a joint arthrodesis for joint stability, but they did not desire a fusion. The patient felt a fusion would limit activities important to her such as texting, playing video games, and applying cosmetics. Joint replacement was offered to the patient as an alternative treatment. In the present case, an HUD was utilized in an off-label manner and inserted into the interphalangeal joint of the patient's thumb. The patient and family were counseled that this was not a standard treatment option and that if fusion was needed in the future, it could be more complicated and require bone grafting compared to using fusion as the initial alternative.

**Figure 3:**
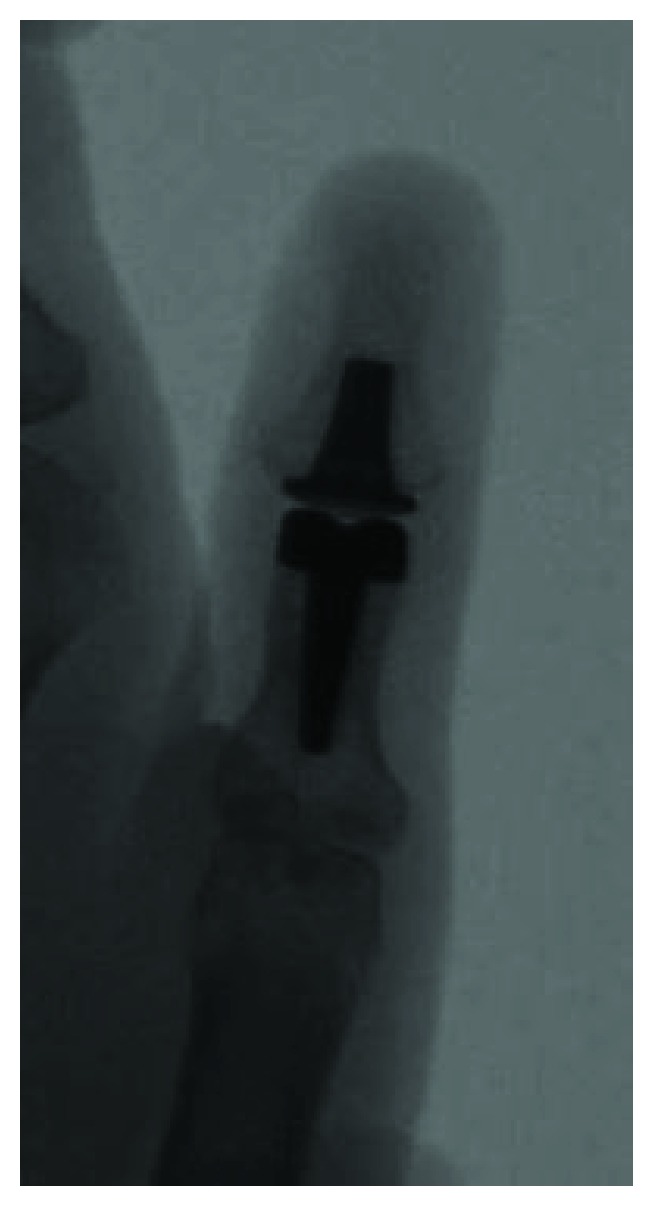
AP view of the thumb IP joint with the implant in place.

**Figure 4:**
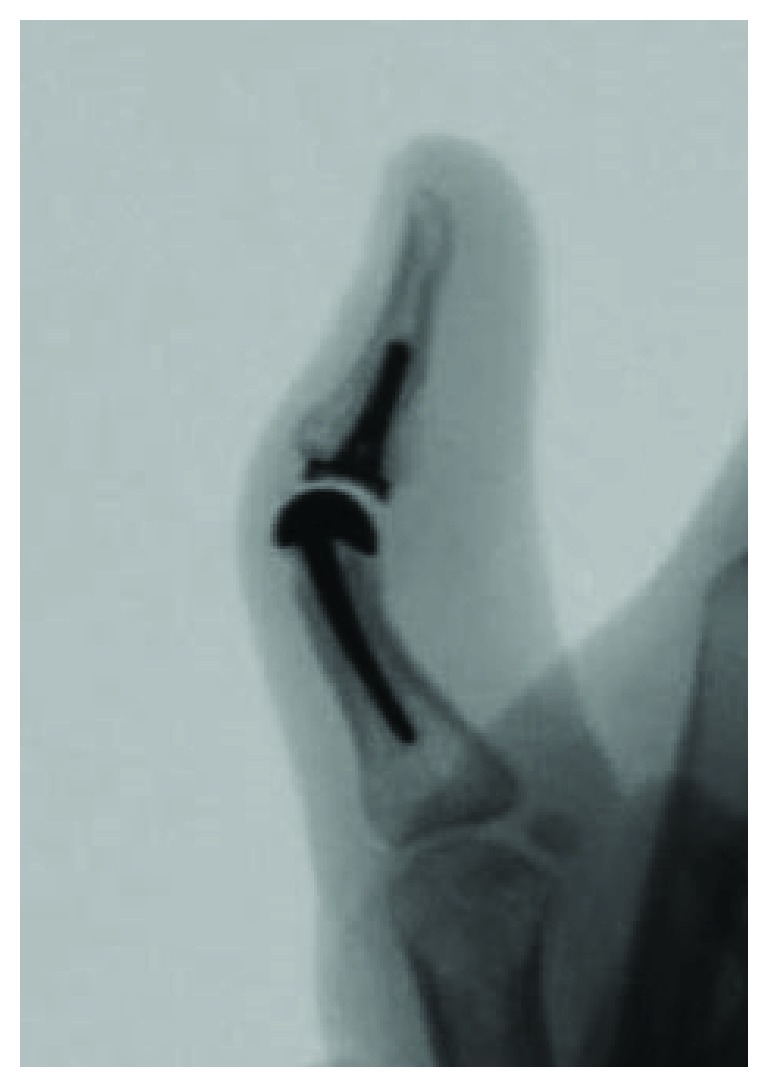
Lateral view of the thumb IP joint with the implant in place.

The patient was followed for 22 months postoperatively and has remained happy with her choice of procedure. The patient's motion in the thumb IP joint is currently a 40 degree arc which is very reasonable given the repair of the flexor pollicis longus tendon and collateral ligament as well. There was excellent range of motion (ROM) in the metacarpophalangeal joint (MCP) which is comparable to the contralateral side. Radiographs demonstrated good seating of the joint implant with no evidence of loosening or periprosthetic fracture (Figures 3 and 4). The collateral ligaments were all stable at the IP joint, and the scar on the left volar thumb was well-healed. The patient reported pain of 0/10 at her most recent follow-up and recorded a DASH score of 6.82. No known complications have arisen as a result of the surgical reconstruction to this point in time.

## 3. Discussion

A recent study demonstrated how the thumb's range of motion impacts certain activities of daily living [8]. It was shown that the position of the thumb influences both the efficiency of the task and the participants' evaluation of the task's ease. Specifically, power tasks were best completed at an angle of 0° for the nondominant hand and an angle between 0° and 30° for the dominant hand. Grip tasks were most efficient at 15° for the nondominant hand and between 0° and 15° for the dominant hand, and pinch tasks were best completed between 0° and 30° for the nondominant hand and between 15° and 30° for the dominant hand. These findings are in accordance with the more flexed posture typically utilized in gripping and strength tasks as these actions are likely to engage the extrinsic flexor pollicis longus (FPL) and increase total muscular involvement.

As previously discussed, arthrodesis of the interphalangeal (IP) joint of the thumb is widely acknowledged as the indicated treatment for trauma and osteoarthritis of this joint [1]. The current standard for interphalangeal joint arthrodesis is to place the thumb in 15-30 degrees of flexion. Given that the efficiency and ease of certain activities of daily living require a range of motion between 0° and 30° [8], it can be assumed that after undergoing arthrodesis, these movements will never be recovered.

Arthrodesis is a standard acceptable method for treatment of damage to the interphalangeal joint of the thumb. It provides a stable, pain-free, motionless joint but can hinder some activities that are an essential part of an individual's activities of daily living. We hypothesized that our subject would benefit from retaining mobility at her thumb interphalangeal joint after joint replacement as she did not prefer fusion. The procedure initially has been a success and is allowing a patient, who is in a crucial developmental life stage, to lead a life closer to preinjury status. Long-term outcomes are unknown, and we will attempt to maintain a long-term follow-up with this patient.

This case is novel as there are no other reported thumb interphalangeal solid joint replacements found in the current literature. The educational value in this report is that it could demonstrate other possibilities and options for surgeons to offer patients who do not wish to move forward with fusion of that joint.

The authors recognize that this was an off-label use of the product in a minor individual. The patient and her parents were engaged in extensive discussions about moving forward with fusion vs. trying the implant. The patient and her mother were well educated about the risks associated with this procedure and understood that replacing the joint in the thumb IP region is not a traditional approach in this situation. Both the patient and her mother were made aware that a fusion may still be necessary in the future. Given the patient's desire to retain motion of her thumb, joint replacement was the only remaining course of treatment.

No adverse effects from the HUD have been noted to this point in time. We hypothesize that future outcomes will be analogous to using the implant in the other digit small joints. The major concern with arthroplasty in any joint is the possible need for revision surgery in the future. The implant that we used is composed of cobalt chrome, titanium, and polyethylene. A similar silicone implant is associated with an 18 percent revision rate in the proximal interphalangeal (PIP) joints [9]. There is low risk of a major adverse outcome associated with this procedure. Patient satisfaction and retained mobility of the joint have been established in this case. We understand that outcomes at 22 months of follow-up are considered short-term in the world of prosthetic joints, and this patient will continue to be closely monitored. A long-term follow-up and future case series will be critical in establishing efficacy and safety.
